# Miscellaneous

**Published:** 1888-01

**Authors:** 


### MISCELLANEOUS.

A Dead Doctrine.—"This doctrine of the damnation of the heathen is dead," writes Rev. Brooke Herford. "It has ceased to be believed in any living sense. People may talk it, but they do not realize what it means to believe it. Why, it is a doctrine which, if those who vote to maintain it had the slightest real sense of what it means, would cast a gloom and shadow over life. What kindly Christian heart that really thought of all that vast ancient world—Egypt, Assyria, Greece, India and China, and all the children of the living God—could think for a moment of their being all in hell, without a sinking of the soul and a doubt whether the whole idea must not be a dreadful dream ? Think of the lofty minds and heroic lives that rose up here and there, like mountain peaks along those far off centuries, so lofty and noble that even yet across the immeasurable years they stand out, visible personalities, Zoroaster and Buddha, and the mild, thoughtful Confucius, and, among the nearer Greeks, many a philosopher and sage who spent his life in the eager striving to discern the truth, and many a hero like those three hundred who fought and died to the last man about Leonidas, their king, to save their country from the mighty hosts of Persia. Nothing but hell for these ? Dare any-one stand up in these days and say squarely that he believes Socrates is in hell ? But it is not of such great ones that I think. Any creed tries to leave some little loop-hole of hope for such as they. The emperor Trajan was believed to be delivered from hell by the prayers of Pope Gregory I ; and Buddha was canonized among the saints, though it is said, only by an inadvertance. But what presses most upon me is the thought of all the nameless myriads who, from the Arctic wastes to the tropical jungles, through all the boundless, unrecorded past, grew up and did the part that was for them in God's world, toiled, loved, fought their fight, and fought it well, reared children and taught them such duty as they knew, and in their rude ways felt after God if happily they might find Him, and, then dimly believing in some greater life to come, passed on to what ? To everlasting hell ? Why, it is horrible ! If men really believe it, it would darken the universe and fill life with gloom. But they do not believe it. The burning of a score of people in a theatre or a railroad car, awakens more real concern in the world in a day than all the flames of hell do in a year. No ! in the common world that idea of there being no hope for any but Christians is utterly dead and gone—and it is not because the common world has grown too little for such a thought to hold its peace, but because it has come to believe too much."

About three years ago the Texas & Pacific Railway Company undertook to sink an artesian well a few miles below Sierra Blanca, which is a little hamlet ninety-five miles east of El Paso. The workmen put the pipe down about six hundred feet when suddenly an underground cavern was struck, the drill dropping about six feet, and a current of air rushed up the pipe. Drilling ceased and the well was abandoned, the six hundred feet of pipe remaining in the ground and giving a connection between the surface of the earth and the strange subterranean cavity a quarter of a mile beneath.

The phenomenon did not at that time attract the attention of any one sufficiently interested to investigate. Recently, however, Superintendent Judy's attention was called to it, and his personal examination and inquiries have developed peculiar facts and testimony about the wonderful well. Governor Brown stopped to see it on his way here. Not many people live near the well, but those who do reside in the neighborhood of it, are thoroughly acquainted with it ever since it was abandoned three years ago. The people near by have been in the habit of going and sitting about the well in summer, to enjoy the cool, invigorating air that rushes up the pipe.

One of the strangest things is the fact that the current of air ebbs and flows like the ocean tides. From about 10:15 A. M. till 10:15 P. M. a current of air rushes out of the pipe with a sound that resembles the noise made by a locomotive "blowing off steam," and so loud that it can be heard for forty or fifty yards. At 10:15 P. M., the overflow air ceases and a strong suction sets in, which lasts for the next twelve hours, this ebb and flow continuing day after day, and it has been observed by horsemen, that, whenever they get in the neighborhood of this well, strong magnetic forces are felt and sparks are given off if the horse's mane is touched.

Recently, a man from Sierra Blanca was sitting close to the well, and on taking out his pocket knife, found a nail which he had in his pocket clinging to the knife. He held the knife in the current of the air and found the magnetic property was greatly increased. Several weeks ago Superintendent Judy held his pocket knife in the current of air for four minutes, and the knife is still strongly magnetized from the effect. The outflowing current of air is believed to possess remarkable curative properties. Its efficacy is to be tested by experiments upon cases of paralysis and other diseases. The people who live near the wonderful well call it the "Fountain of Youth."

Drooping Shoulders.—This is a serious evil. It compromises both appearance and vitality. A stooping figure is not only a familiar expression of weakness or old age, but is, when caused by careless habits, a direct cause of contracted chest and defective breathing. Unless you rid yourself of this crook while at school, you will probably go bent to the grave. There is one good way to cure it. Shoulder-braces will not help. One needs, not an artificial substitute, but some means to develop the muscles whose duty it is to hold the head and shoulders erect. I know of but one bull's eye shot. It is to carry a weight on the head. A sheep-skin or other strong bag filled with twenty to eighty pouuds of sand is a good weight. When engaged in your morning studies, either before or after breakfast, put this bag of sand on the head, hold your head erect, draw your chin close to your neck, and walk slowly about the room, coming back, if you please, every minute or two to your book, or carrying the book as you walk. The muscles, whose duty it is to hold head and shoulders erect are hit, not with scattering shot, but with a rifle ball. The bones of the spine and intervertebral substance will soon accommodate themselves to the new attitude. One year of daily practice with the bag, half an hour morning and evening, will give you a noble carriage, without interfering a moment with your studies.

It would be very difficult to put into a paragraph more important instructions than this. Your respiration, voice and strength of spine, to say nothing of your appearance, will find a new departure in this cure of drooping shoulders.—*Dr. Dio Lewis*

COLOR BLINDNESS CURED.—Color blindness has usually been supposed to be incurable. Dr. Babbitt, Dean of the New York College of Magnetics, 39 West 27th St., New York, believes he can cure every case. He declares that Sir David Brewster, Tyndall, Chevreul, Helmholtz and the other savants are absolutely wrong in their philosophy of vision, all color forces being received by the retina on principles of chemical affinity, which principles as applied to sunlight are totally unknown to our scientists.

We have the very best accounts of Carl J. Jensen's Pepsin, as not only an aid to impaired digestion, but as a cure for catarrhal affections of long standing. This, of all seasons, is the one when it is most needed. Headquarters, 161 W. 23rd Street New York City, and 2039 Green St. Philadelphia.

WARNER'S LOG CABIN REMEDIES—old-fashioned, simple compounds, used in the days of our hardy forefathers, are "old timers," but "old reliable." They comprise a "Sarsaparilla," "Hops and Buchu Remedy," "Cough and Consumption Remedy," "Scalpine, for the Hair," "Extract," for External and Internal use, "Plasters," "Rose Cream," for Catarrh, and "Liver Pills." They are put up by H. H. Warner & Co., proprietors of Warner's Safe Remedies, and promise to equal the standard value of those great preparations. All druggists keep them.

### ROSES.

### I.

IN THE GARDEN.

Hair of raven hue in twining curls,
Lips like rubies bright,
Teeth like shining pearls,
Two roguish dimples in two rosy cheeks,
That laugh and twinkle
While my darling speaks,
Two little feet that scarcely, as she walks
O'er dewy grass and flowerets,
Incline their tender stalks.
Such is my loved one, roaming here with me
Mid violets and roses, far less fair than she.

### II.

AT THE BALL.

n wavy tresses flows her hair to-night
O'er eyes, that drooping 'neath my gaze,
Shine full of tender light,
As through the envious lace, but half revealed
A bosom, that would Juno grace,
Gleams whitely; half concealed,
One flower alone, my gift, lies there enshrined,
That in such resting place should be
Most envied of its kind.
Such is my darling, of all blossoms there
My Rose is fairest of those flowerets fair.

LORING G. BARRY.

